# The Treatment Landscape of Elderly Patients with Hormone Receptor-Positive Her2 Negative Advanced Breast Cancer: Current Perspectives and Future Directions

**DOI:** 10.3390/jcm12186012

**Published:** 2023-09-16

**Authors:** Carmelo Laface, Francesco Giuliani, Assunta Melaccio, Maria Nicla Pappagallo, Anna Natalizia Santoro, Martina Perrone, Pierluigi De Santis, Chiara Guarini, Daniela Carrozzo, Palma Fedele

**Affiliations:** 1 Medical Oncology, Dario Camberlingo Hospital, 72021 Francavilla Fontana, Italyannanatalizia.santoro@asl.brindisi.it (A.N.S.); martina.perrone@asl.brindisi.it (M.P.); pierluigidesantis@asl.brindisi.it (P.D.S.); chiara.guarini@asl.brindisi.it (C.G.); daniela.carrozzo@asl.brindisi.it (D.C.); 2 Medical Oncology, San Paolo Hospital, ASL Bari, 70123 Bari, Italy; francesco.giuliani@asl.bari.it (F.G.); assunta.melaccio@asl.bari.it (A.M.); maria.pappagallo@asl.bari.it (M.N.P.)

**Keywords:** elderly, breast cancer, endocrine therapy, targeted agents, comprehensive geriatric assessment

## Abstract

Breast cancer (BC) in elderly women is an increasing health issue due to demographic changes. BC tends to present later and may receive less than standard treatment options. More often, BC in elderly patients is endocrine-positive (HR+). The treatment of elderly patients with metastatic BC (mBC) represents a therapeutic challenge. In recent years, the treatment landscape of patients that are HR+/Her2-negative has changed due to the introduction in clinical practice of new targeted drugs, which have improved patient outcomes. Elderly patients are a small percentage of all patients enrolled in clinical trials and, to date, there are no standardized guidelines that define the best treatment option for this patient population. This can lead to undertreatment or overtreatment, impacting patient morbidity and mortality. Geriatric Assessment tools to tailor the treatment in elderly patients are underused because they are long and difficult to apply in a busy routine clinical practice. For all these reasons, there is an urgent need to produce data about the best treatment for elderly patients with HR+ mBC. Herein, we report data from randomized clinical trials and real-world evidence on the therapeutic options for HR+ Her2-negative mBC elderly patients and explore future treatment directions.

## 1. Introduction

In the last century, all the industrialized countries have witnessed an impressive lengthening of the average life span. Life expectancy was just over 40 years at the end of the 19th century, while it is about double nowadays. Furthermore, the over-60s currently have a life expectancy of 24 years, and the over-85s of 6 years. This improvement determines the progressive increase in the number of elderly people in the overall population [1]. With advancing age, the risk of cancer increases; in fact, more than 50% of solid tumors (breast, prostate, lung, colon cancers) are diagnosed in patients over the age of 65 [2,3]. It has been estimated that in 2030, 70% of all cancers will affect patients aged over 65 years in the USA [4]. Management of cancer in the elderly population is a challenge, because of the clinical complexity of these patients. In this regard, treatment decisions are based on chronological age, but this does not reflect the heterogeneity of this population and predicts poor treatment tolerance. Different pieces of evidence suggest that the decision-making process should be based on the patient’s function rather than on chronological age [5]. For this reason, cancer patients over the age of 70 must undergo a multidimensional geriatric evaluation [6,7]. Evaluating the elderly before subjecting them to cancer therapy would allow physicians to make more weighted choices.

Breast cancer is a disease of aging [8,9]; in fact, about 30% of BCs are diagnosed in patients aged older than 70. BC mortality rate is decreasing due to screening programs and the introduction of new active treatments [10], but this is not true for elderly patients [11]. Elderly BC patients are usually diagnosed in the late stages of the disease for different reasons: patient delay, higher prevalence of fatalistic view, lower awareness of BC, fear, and anxiety of treatment. More often, mBC in elderly patients is endocrine-positive (HR+), characterized by a higher percentage of hormone receptor (HR) expression and a lower expression of human epidermal growth factor receptor 2 (HER2). Moreover, elderly mBC patients are less likely to receive standard treatments (hormone therapy, chemotherapy, radiotherapy) because of comorbidities, limited life expectancy, assumptions that BC is less aggressive, and decisions based on the toxicity profiles of drugs, as well as financial and cultural reasons.

The estrogen receptor (ER) acts an important driver in the tumorigenesis, proliferation, and progression of BCs. Targeting ER signaling at different levels has proven to be a successful therapeutic strategy in the control of HR+ BC [12]. Endocrine therapy has been the standard therapy for HR+ BC in the early and advanced stages with several agents, such as selective estrogen receptor modulators (SERMS), aromatase inhibitors (AIs) and selective estrogen receptor degraders (SERDs) [12]. Adjuvant hormone therapy is the treatment of choice for nearly all patients with HR+ BC to prevent local–regional recurrence, metastatic disease, and contralateral tumors [13]. Tamoxifen, an SERM, for five years has been the standard of care, regardless of menopausal status [13]. Another option regards AIs that block the conversion of androgens into estrogens, suppressing residual estrogen levels by more than 90% in postmenopausal women [14]. These agents are contraindicated in premenopausal women who are not undergoing ovarian suppression, because compensatory physiological responses induce ovarian estrogen production. AI therapy results in a greater reduction in the risk of recurrence than 5 years of tamoxifen, such that most postmenopausal women should consider aromatase inhibitor treatment either as initial therapy or after 2 to 3 years of tamoxifen [14]. Extending the duration of treatment from 5 to 10 years with either tamoxifen [15] or aromatase inhibitors [16] reduces, although only mildly, the risk of recurrence, as compared with just 5 years of treatment [17]. Endocrine-based therapy is the standard of care as the initial therapy for metastatic disease, except in patients with markedly symptomatic BC and visceral crisis, which warrant initial chemotherapy [18]. The selection of endocrine agents is governed by the prior adjuvant therapy, if administered. Premenopausal women with advanced ER-positive cancer should undergo ovarian suppression, which improves survival. Treatment with an AI or tamoxifen is effective in controlling advanced disease [18]. Fulvestrant, an SERD that binds to ER and functionally eradicates the receptor, is active in tumors that are refractory to tamoxifen or aromatase inhibitor therapy [19]. Due to the high degree of efficacy and wide therapeutic indices of endocrine therapies, patients may receive such treatments for progressive disease over the course of several years [20]. Unfortunately, the majority of ER+ metastatic breast cancers that initially respond to endocrine treatment will become refractory despite continued ER expression [20]. Acquired resistance to endocrine therapy remains a significant clinical burden for BC patients. Somatic mutations in the ESR1 (estrogen receptor alpha (Erα)) gene ligand-binding domain (LBD) represent a recognized mechanism of acquired resistance [20]. Somatic mutations to ESR1 LBD were identified in 25–30% of patients who previously received endocrine treatment [21,22,23], with no difference according to age [24]. ESR1 mutations induce constitutive ER activity, in turn upregulating ER-dependent gene transcription and causing resistance to estrogen-depleting therapies [25]. Some studies have been evaluating the safety and efficacy of different hormone agents in those patients who previously received endocrine therapies and developed ESR1 mutations. For example, the ELAINE-1 trial randomized women with locally advanced/metastatic ER+/HER2− BC, an ESR1 mutation, and disease progression on AI and cyclin-dependent kinase 4/6 (CDK4/6) inhibitors, to receive lasofoxifene vs. fulvestrant. All the results numerically favored the experimental group. These data must be confirmed in a larger, adequately powered clinical study.

In recent years, the treatment landscape of HR+/Her2-negative mBC has completely changed, thanks to the introduction of particularly active targeted drugs, from CDK4/6 inhibitors [26,27,28,29,30,31,32], mTOR inhibitors [33] and PARP inhibitors [34,35], to new oral SERDs [36,37,38] and PI3K inhibitors [39,40,41,42], which have expanded the treatment armamentarium and improved survival outcomes for this patient population. Moreover, other new active drugs are increasingly appearing in the setting of HR+ mBC, such as antibody–drug conjugates (ADC) [43,44], which have recently demonstrated their clinical efficacy regardless of the expression of HRs, PROteolysis Targeting Chimeras (PROTACs) [45], selective estrogen receptor covalent antagonists (SERCA) [46] and complete estrogen receptor antagonists (CERAN) [25]. However, there are many concerns about the application of these new treatment strategies to elderly or unfit patients due to the possible risk of adverse events (AEs) and/or adherence and compliance issues of elderly patients to the treatment [47,48]. Moreover, to date, there are no prospective randomized studies on the efficacy and safety of cancer treatments (chemotherapy and targeted therapy) on patients over 70 years; most of the evidence for this subgroup of patients derives from exploratory analyses of randomized studies and real-life retrospective or prospective studies. Here, we report an analysis of the currently available evidence on the therapeutic options and on the future perspectives of treatment for elderly HR+ mBC patients.

## 2. Patient Selection and Geriatric Evaluation

A comprehensive evaluation of an elderly patient before initiating active cancer treatment is recommended [49]. In particular, the elderly patient should be evaluated within an interdisciplinary team to distinguish suitable patients who may be candidates for cancer treatments from prefrail or frail patients for whom cancer treatments could have negative effects on quality of life (QoL) or survival [50]. In addition, other aspects to consider when appointing an elderly patient to cancer therapies are life expectancy and cognitive status, as well as the polypharmacy, comorbidities, and potential AEs of cancer therapies. Multidimensional geriatric assessment is essential for screening patients who may receive specific cancer therapies and identifying those patients to refer to the geriatric palliative care setting [51,52,53]. In this regard, the International Society of Geriatric Oncology (SIOG) suggests that elderly patients should undergo a validated comprehensive geriatric assessment (CGA) [54]. The CGA is a multidisciplinary tool used to explore the impact of age-associated physiologic factors, in contrast with chronologic age, that may affect health and disease in older patients [55,56,57].

CGA consists of a series of validated instruments that can be used to evaluate the impacts and needs under different domains, as regards functional status, cognition, comorbidities, polypharmacy, psychosocial function, social support, and nutritional status. All these items can affect outcomes in older patients with cancer. Classical CGA classifies patients in good general health as suitable, those with partial impairment in some domains as vulnerable, and those with severe impairment in most domains as fragile [58]. This allows the identification of patients at risk of side effects, and can possibly guide management, treatment, and follow-up.

However, CGA is underused because it is long and difficult to apply in a busy routine clinical practice. In this regard, there are other tools for evaluating the frailty of elderly patients that help in the choice of treatment, such as the Cancer and Aging Research Group-Breast Cancer (CARG-BC) score [59]. This is constructed by combining eight clinical and geriatric variables (stage (II/III), planned anthracycline-based regimen, planned duration of treatment (>3 months), abnormal liver function, anemia (hemoglobin Male ≤ 13/Female ≤ 12 g/dL), ≥1 fall in the past 6 months, limited ability to walk more than 1 mile, and lack of someone to give good advice in a crisis), and was developed to classify older patients with early-stage BC into low, intermediate, and high risk for grade 3–5 chemotherapy toxicity. The score was externally validated; demonstrated to better predict toxicity compared with prior models and physician-rated performance status; and was strongly associated with dose reductions, dose delays, early treatment discontinuation, reduced dose intensity, and hospitalizations [59].

## 3. Treatment of Elderly Metastatic HR+ Breast Cancer Patients with Targeted Therapies

HR+ or Luminal BC is the most frequently diagnosed subtype in elderly patients [60]. Despite a third of BCs being diagnosed in patients older than 70, elderly patients are underrepresented in clinical trials [52,61,62]. As a result, we are missing prospective randomized data on optimal treatment strategies in this subset of patients and therapeutic guidelines. Furthermore, there is no consensus on the definition of “elderly patient”. In this regard, many clinical studies consider the age of 65 as a cut-off, while according to more recent data, it would be more appropriate to consider patients elderly from 70 years old [63]. In the last few years, many targeted drugs have been developed for the treatment of HR+ Her2-negative mBC, especially to overcome endocrine resistance.

### 3.1. Everolimus

Everolimus is the first-in-class targeted agent to reach the oncology clinic. BOLERO-2 is a phase III randomized trial comparing everolimus plus exemestane versus exemestane alone in 724 postmenopausal women with HR+ mBC after treatment with nonsteroidal aromatase inhibitors. In the study, 164/724 patients were older than 70 years. The primary endpoints were progression-free survival (PFS) (primary endpoint) and overall survival (OS), while response rate (RR) and safety were secondary endpoints [64]. In the study, the combination of everolimus plus exemestane was effective in elderly patients, and PFS has also improved in this subgroup of patients (hazard ratio, 0.59 (≥65 years) and 0.45 (≥70 years)) [65].

### 3.2. CDK 4/6 Inhibitors

CDK 4/6 inhibitors are standard of care as the first- or second-line treatment in endocrine-sensitive or endocrine-resistant mBC. Most of the published analyses demonstrate that the combination of hormone therapy plus CDK 4/6 inhibitors versus hormone therapy alone leads to a survival advantage even in the subgroup of patients aged >65 years [66]. In the three Paloma trials that explored the efficacy and safety of Palbociclib ± endocrine therapy in hormone-sensitive [27] and hormone-resistant mBC patients [28,67], only 37% of the enrolled population were between 64 and 75 years old, while only 9% of the patients were over 75 years old. Efficacy data in the elderly subgroup are consistent with the results of the general population, and demonstrate a better PFS in the experimental arm compared to endocrine therapy alone. However, over 75 patients were poorly represented in this trial, as well as in other in clinical studies, and therefore it is not possible to draw definitive considerations on this patient subgroup. In Paloma 1 e 2 trials, PFS was 22 months in the 162 patients who received Letrozole plus Palbociclib versus 12.3 months in Letrozole as single agent (HR (CI 95%) 0.66 (0.40–0.64); *p* < 0.001)) in the 65–74 years subgroup of patients. In the Paloma 3 trial, 59 patients between 65 and 74 years received the combination of Palbociclib and Fulvestrant. PFS was 16.1 months versus 3.7 for Fulvestrant alone (HR (CI 95%) 0.27 (0.16–0.48); *p* < 0.001)) [68]. The same results have been shown in the retrospective analyses of PFS regarding elderly patients enrolled in the Monaleesa trials. PFS was Not Reached (NR) versus 18.4 months in 150 patients >65 years who received Ribociclib plus Letrozole versus Letrozole alone in the Monaleesa 2 trial (HR (CI 95%) 0.61 (0.39–0.94)) [69]. In the Monaleesa 3 trial, HR for PFS in patients older than 65 was 0.59 (0.43–0.81) in the combination arm versus 0.69 (0.45–0.81) in the Fulvestrant alone group [30]. In the Monarch 2 trial, 114 patients were over 65 and received Abemaciclib + Fulvestrant, obtaining a PFS of 14.4 months (HR (CI 95%) 0.63 (0.43–0.94)) versus 8.1 months in the 60 patients in the Fulvestrant alone group [70]. In the Monarch 3 trial, 106 patients were over 65 years and received the combination of Abemaciclib plus Aromatase inhibitors. PFS was 28.2 months versus 24.2 months in the 54 patients that received aromatase inhibitor alone (HR (CI 95%) 0.63 (0.43–0.94)) [70]. In the Paloma 1 and 2 trials, PFS was NR in the 56 patients over 75 who received Letrozole plus Palbociclib versus 10.9 months in the 26 patients in the Letrozole alone group (HR (CI 95%) 0.31 (0.16–0.61) *p* < 0.001) [27,67]. In the Paloma 3 trial, only 27 patients over 75 received Palbociclib plus Fulvestrant and reported a median PFS of 13.6 months versus the 7.4 months for the 6 patients that received Fulvestrant alone (HR 0.59 (CI 95%) 0.19–1.80) *p* < 0.18)) [28]. No data are available on the outcomes of the patients over 75 enrolled in the Monaleesa trials [30,69]. In the Monarch 2 trial, patients over 75 numbered 41 in the Abemaciclib + Fulvestrant group versus 39 in te Fulvestrant alone group, and they reported a median PFS of 13.9 months versus 5.8 months (HR (CI 95%) 0.62 (0.34–1.11)) [32]. In the Monarch 3 trial, 42 patients received Abemaciclib plus Aromatase inhibitor and only 20 received Aromatase inhibitor alone. The median PFS values were, respectively, 31.1 versus 9.1 months (HR (CI 95%) 0.54 (0.26–1.13)) [31]. Several retrospective and prospective real-life studies have evaluated the safety and efficacy of CDK4/6 inhibitors in elderly patients (Table 1). The PFS data are consistent with those of the randomized trials. Among the real-life studies, Palomage was the first to prospectively evaluate the feasibility of Palbociclib plus endocrine therapy in a cohort of patients over 70 years [71]. In this study, 40% of the patients were ≥80 years old, and 20% of them had an ECOG performance status ≥2. In total, 70% of the patients had undergone a multidimensional geriatric assessment, and 30% of them had some level of vulnerability. Although the safety of CDK4/6 inhibitors in older patients is consistent with that of younger patients, a higher incidence of grade 3 and 4 AEs was reported in the elderly ones, particularly for the over-75s. From the published studies about CDK4/6 inhibitors, it does not appear mandatory to practice an upfront dose reduction in elderly patients based only on age [72].

### 3.3. PI3K Inhibitors

Alpelisib is a PI3Kα-specific inhibitor that has shown, in the phase III Solar1 trial, a better PFS compared to endocrine therapy alone in mBC patients who had received previous endocrine therapy [39]. In particular, the combination of Alpelisib plus Fulvestrant was particularly active in the cohort of PIK3CA-mutated patients: PFS was 11.0 months (95% confidence interval (CI), 7.5 to 14.5) in the Alpelisib–Fulvestrant arm versus 5.7 months (95% CI, 3.7 to 7.4) in the placebo–Fulvestrant arm (HR for progression or death, 0.65; 95% CI, 0.50 to 0.85; *p* < 0.001). In the study, 117/285 patients who received Alpelisib were over 65 years of age, and 34 patients were between the ages of 75 and 87 years old. No overall differences in PFS outcome and Alpelisib exposure were observed between these patients and younger patients. However, grade 3 or 4 hyperglycemia, an AE related to Alpelisib, was reported more frequently in patients over 65 years of age (44% vs. 32%).

### 3.4. PARP Inhibitors

Two different PARP inhibitors, Olaparib and Talazoparib, have been approved for the treatment of mBC harboring BRCA mutations, based on the results of the OlympiAD [34] and EMBRACAs studies [35]. In the OlympiAD trial, only 15/302 patients were older than 65 years: 11 received Olaparib, and 4 received the standard therapy. Similarly, in the EMBRACA trial, patients over 65 comprised 37 of the 431 enrolled; 27 (9.4%) of them received Talazoparib, 10 (6.9%) received the standard therapy. Given the small number, a statistical analysis on this subgroup of patients was not carried out.

### 3.5. Elacestrant

Among all the oral SERDs, Elacestrant is the first-in-class. The Emerald trial enrolled mBC patients who previously had received one to two lines of endocrine therapy, a CDK4/6 inhibitor, and one or fewer lines of chemotherapy. The study randomized patients to Elacestrant 400 mg or a treatment of investigator’s choice (aromatase inhibitor or Fulvestrant) [36]. The median PFS values were 2.79 and 1.91 months with Elacestrant and standard therapy in the intent-to-treat population, respectively (HR, 0.697; 95% CI, 0.552–0.880; *p* = 0.0018)). In particular, in tumors harboring ESR1 mutations, PFS was 3.78 months vs. 1.87 months with standard therapy, leading to a 45% reduction in the risk of disease progression or death in this subgroup (HR, 0.546; 95% CI, 0.387–0.768; *p* = 0.0005). In the Emerald trial, 215/477 patients were over 65 years old. No differences were found in terms of efficacy in patients aged ≥65 years compared to younger ones.

### 3.6. Capivasertib

Capivasertib is a potent, selective, adenosine triphosphate (ATP)-competitive inhibitor of all three AKT isoforms (AKT1/2/3). The phase III double-blind, randomized, Capitello-291 trial evaluated Capivasertib in combination with Fulvestrant versus placebo plus Fulvestrant for the treatment of pretreated locally advanced or metastatic HR+, HER2-low, or BC-negative patients. PFS, the primary endpoint of the study, was significantly improved in the experimental group, including those with AKT pathway-altered tumors (7.2 months (95% CI, 7.2–7.4) vs. 3.6 months (95% CI, 2.8–3.7; HR, 0.60; 95% CI, 0.51–0.71; 2-sided *p* < 0.001)). In the study, 217/708 patients were over 65 years. The PFS benefit derived from Capivasertib plus Fulvestrant was consistent across age subgroups (in ≥65: HR, 0.65; 95% CI, 0.47–0.90; in ≤65: HR, 0.65; 95% CI, 0.53–0.79) [78].

## 4. Treatment of Elderly HR+ Metastatic Breast Cancer Patients with Chemotherapy

Chemotherapy is usually indicated in elderly patients with hormone receptor-negative disease or receptor-positive disease resistant to endocrine therapy, or with rapidly progressive or extensive visceral disease [5].

However, this patient subgroup represents a less likely candidate for chemotherapy due to the increased risks related to chemotoxicity, comorbidities, and life expectancy [79]. To prevent chemotoxicity, toxicity calculators (e.g., CARG and CRASH) have been formulated. The CARG model considers patient, tumor, and chemotherapy characteristics, geriatric assessment variables, and laboratory test values, and is able to predict the risk of developing grade 3 to 5 AEs from cancer treatment. In the CRASH model, the ECOG performance status, the Mini Nutritional Assessment, the Mini-Mental State Examination, laboratory tests, and the type of chemotherapy administered are considered [80]. However, these scores cannot be used as an exclusive tool to decide whether to treat one patient with chemotherapy, and less so to identify which drug to use. For example, single-drug regimens are preferred over polychemotherapy, and in this contest, drugs that have already been investigated in older populations should be selected. In addition, changes in treatment schedules, dose reductions, or dose escalations before reaching the standard recommended dose can help to reduce AEs [5].

For patients with hormone-sensitive mBC who have become refractory to hormone therapy, sequential chemotherapy is considered the best option [81]. Unfortunately, few studies have investigated the use of chemotherapeutic agents in the elderly. Eribulin mesylate (Halaven), a synthetic analog of halichondrin B, is a non-taxane microtubule inhibitor that acts differently with respect to other tubulin-targeting agents and overcomes resistance to paclitaxel [82,83]. The Phase III, open-label, randomized, global, multicenter EMBRACE trial (Eribulin alone versus treatment of physician choice in patients with mBC) allowed the approval of Eribulin in patients with local or mBC pretreated with at least an anthracycline and a taxane [84]. Two other single-arm phase II open-label studies, 201 and 211, involved patients with locally advanced BC or mBC pre-treated with anthracycline, taxanes, or capecitabine. These studies confirmed the efficacy of Eribulin in terms of OS, PFS, RR, and clinical benefit rates (CBR) [85,86]. Study 201, Study 211, and EMBRACE demonstrated that Eribulin is a usable drug after treatment with anthracyclines or taxanes, with similar efficacy and toxicity regardless of age, and with no effect on geriatric scores or QoL [5]. Muss et al. conducted an exploratory analysis based on three randomized trials to compare the efficacy and tolerability of Eribulin between young patients and patients older than 70 years. Eight hundred and twenty-seven patients received Eribulin mesylate at 1.4 mg/m^2^ as two to five small intravenous infusions on days 1 and 8 of a 21-day cycle. The results of this analysis establish that age did not influence the OS, PFS, ORR, CBR, or tolerability of Eribulin as a single agent [87]. In 2019, a multicenter observational study based on geriatric oncology parameters (sponsored by GIOGer, Italian Group of Geriatric Oncology) was conducted on 50 elderly patients with mBC to evaluate the efficacy and tolerability of Eribulin. Italian versions of the CGA Item Score and Health-Related Quality of Life (HRQL) score were used for assessment. The study demonstrated that treatment with Eribulin succeeded in preserving the QoL and geriatric parameters included in the CGA, except instrumental functioning and geriatric depression [88].

Among single-drug regimens, weekly administered taxanes, such as paclitaxel and docetaxel, are some of the most widely used drugs in older patients with mBC [5]. However, albumin-bound paclitaxel (nab-paclitaxel) is much less neurotoxic than these two solved-based taxanes, it does not require steroid premedication, and is associated with a lower rate of hypersensitivity reaction [89,90,91].

The randomized phase II EFFECT study determined the optimal weekly dose of nab-paclitaxel for elderly patients with mBC by geriatric assessment. The study showed that the 100 mg/m^2^ dose (arm A) on days 1, 8 and 15 of a 28-day cycle, was significantly better tolerated than the 150 mg/m^2^ dose (arm B) in 160 randomized women with mBC aged ≥65 years. Neurotoxicity-related events were also lower [92].

## 5. Treatment of Elderly HR+ Metastatic Breast Cancer Patients with Antibody–Drug Conjugates

Antibody–drug conjugates (ADCs) consist of an antibody targeting a specific antigen and a toxic payload joined by a linker. ADC components ensure the maximum delivery of the chemotoxic drug to tumor cells while preserving healthy tissue and reducing off-target toxicity [93].

Currently, in the BC treatment, there are three ADCs already used in clinical practice, and others are very promising in the experimental stage.

Trastuzumab emtansine (T-DM1) is the first ADC to have been approved for the treatment of patients with resectable or HER2-positive mBC previously treated with trastuzumab and taxane, or who relapsed during or within 6 months of completed adjuvant therapy with trastuzumab [94]. It targets the HER2 receptor of trastuzumab with the cytotoxic activity of the microtubule inhibitory agent DM1 [94]. Thanks to the KATHERINE T-DM1 study, early HER2-positive BC also obtained approval in the adjuvant treatment of patients with residual invasive breast and/or lymph node disease after neoadjuvant treatment with at least one taxane and trastuzumab-based treatment regimen [95].

The DESTINY-Breast04 study evaluated Trastuzumab-deruxtecan (T-dxt), an antibody–drug conjugate consisting of a humanized anti-HER2 monoclonal antibody linked to a topoisomerase I inhibitor payload through a tetrapeptide-based cleavable linker. The study led to the approval of this drug for the treatment of patients with unresectable or metastatic HER2-low BC, who have received prior chemotherapy for metastatic disease or who developed disease recurrence during or within 6 months of completion of adjuvant chemotherapy [43].

A sub-analysis of this study was conducted, and the results were presented at the 2022 San Antonio Breast Cancer Symposium. It considered the patient’s history and the characteristics of the disease. Notably, patients 65 years of age and older had a higher median PFS with Trastuzumab deruxtecan compared with those treated with TPC (11.4 months vs. 6.2 months; HR, 0.57; 95% CI, 0.36–0.89), and those younger than 65 had a median PFS of 9.8 months vs. 4.6 months, respectively (HR, 0.47; 95% CI, 0.37–0.61) [43,96].

Sacituzumab Govitecan (SG) is the latest ADC demonstrating promising activity in the phase I/II IMMU-132-01 basket study conducted in heavily pretreated solid tumors, including HR+/HER2− and triple-negative mBC. The drug consists of a trophoblast cell surface antigen 2 (Trop-2) antibody linked to a very potent and active metabolite of irinotecan (SN-38) via a hydrolyzable linker [44,97]. SG initially received breakthrough designation from the US FDA in patients with triple-negative BC who had failed prior therapies for metastatic stage based on the durable RR of 33% by local assessment with a safety profile that was manageable in this patient setting [98]. The multicenter, randomized phase III ASCENT study (NCT02574455) confirmed these results [99]. The pivotal, randomized, multicenter, phase III TROPiCS-02 study investigated whether SG monotherapy can improve PFS and ORR in HR+/HER2 mBC patients with one or more prior endocrine therapy and two to four lines of chemotherapy compared to standard TPC chemotherapy [44]. The recent results of this study confirm the superiority of SG in terms of clinically significant OS in pretreated, endocrine-resistant, HR+/HER2–mBC patients regardless of HER2 status [100]. The efficacy and safety results were consistent across age subgroups. In the study, 140 patients were over 65 years and reported a median PFS of 6.7 (4.2–9) months with SG vs. 3.5 (1.7–5.6) months with single-agent chemotherapy (HR, 0.59; 95% CI, 0.38–0.93).

New ADCs are currently under investigation, such as Trastuzumab duocarmazine (also known as SYD985), Disitamab vedotin (also known as RC48-ADC), and Ladiratumab vedotin (also known as SGN-LIV1A); ADCs are also being studied in earlier lines of treatment in BC and other tumor entities, as a single agent or in combination with checkpoint inhibitors, tyrosine kinase inhibitors, and the combination of treatments directed against HER2 [93].

## 6. Future Perspectives

The percentage of elderly patients is increasing and will represent an important proportion of patients with mBC soon. BC in elderly patients seems to have different biological characteristics compared to younger patients. The adequate evaluation of the biological characteristics of the disease, through gene array techniques, is one of the fundamental aspects of applying precision medicine and avoiding undertreatment or overtreatment. The treatment landscape of HR+ mBC is rapidly changing, and new active drugs will soon join the currently available therapeutic armamentarium. CDK4/6 inhibitors are the standard of care as first-line therapy in patients with HR+ mBC. There are no studies that directly compare the CDK 4/6 inhibitors, such as Palbociclib, Ribociclib and Abemaciclib, and therefore it is not possible to demonstrate the superiority of one drug over the other two. Therefore, the choice of the most suitable treatment must be based on several factors: patient characteristics (performance status, compliance, comorbidities); drug characteristics (pharmacokinetic and pharmacodynamic properties); data from clinical trials, and patient preference. Since the efficacy and safety of CDK4/6 inhibitors in elderly patients are comparable to those in younger patients, undertreatment or upfront dose reduction based on chronological age should be discouraged. The choice of CDK4/6 inhibitor should take particular account of possible drug interactions in those patients with multiple comorbidities and polypharmacotherapy.

To date, there is no standard second-line treatment for patients who have experienced disease progression after first-line CDK4/6 inhibitors ± endocrine therapy. However, the efficacy data on the new drugs (Elacestrant, Capivasertib, Alpelisib), even in previously treated patients over 65 years of age, encourage us to also offer a targeted option in the second line of therapy, delaying the use of chemotherapy to subsequent lines of treatment. Moreover, the oral route of administration of this new drug could preserve the QoL of elderly patients, avoiding the morbidity associated with long-term central venous access and reducing the utilization of healthcare facilities. 

Chemotherapy and new ADCs should be reserved for those patients who progress to endocrine therapy ± targeted agents, or for elderly women with rapidly progressing mBC, with a preference for mono-, oral and weekly chemotherapies.

Although endocrine therapies play a fundamental role in the treatment of patients with HR+ BC, most patients with advanced disease will develop resistance [101]. Aromatase inhibitors block the conversion of androstenedione to estrogen, increasing the concentration of androgens that could then stimulate the androgen receptor (AR) [102,103], which is expressed in >75% of HR+ BC [104,105]. The role of the AR in BC has not been well defined yet; it has been associated with better patient outcomes in patients with HR+ disease [106,107], but has also been associated with resistance to endocrine therapies [108,109]. Enzalutamide is a potent inhibitor of AR signaling [110,111,112,113,114]; in preclinical BC models, enzalutamide blocked both the estrogen- and the androgen-mediated growth of HR+ cells [108,109]. These results suggest blocking both AR and ER signaling in patients with HR+ BC could provide additional benefits beyond ER inhibition alone, and may prevent resistance. A phase II trial by Krop et al. evaluated the efficacy, safety, and predictive biomarkers of enzalutamide plus exemestane in women with advanced HR+ BC [115]. PFS was not improved in either cohort of the ITT population. In cohort 1 (patients with no prior endocrine therapy), high levels of AR mRNA were associated with greater benefits related to enzalutamide. This effect was particularly apparent in patients with both high levels of AR mRNA and low levels of ESR1 mRNA (HR, 0.24 (95% CI, 0.10–0.60); *p* = 0.0011). Enzalutamide with exemestane was well tolerated. On these bases, endocrine therapy-naïve patients with high AR mRNA levels, especially in combination with low ESR1 mRNA levels, may benefit from the addition of enzalutamide to exemestane. Another phase II clinical trial tested the combination of fulvestrant with enzalutamide in heavily pretreated women with advanced ER+/HER2− BC [116]. The median PFS was 8 weeks (95% CI: 2–52). AEs were as expected for endocrine therapy. Significant (*p* < 0.1) univariate relationships existed between PFS and ER%, AR%, and *PIK3CA*, and/or *PTEN* mutations. Baseline phospho-proteins in the mTOR pathway were more highly expressed in biopsies of patients with shorter PFS. The primary endpoint of clinical benefit rate at 24 weeks was 25% in heavily pretreated advanced ER+/HER2− BC. Short PFS was associated with the activation of the mTOR pathway, and *PIK3CA* and/or *PTEN* mutations were associated with an increased hazard of progression. Thus, a combination of fulvestrant or other SERD plus the AKT/PI3K/mTOR inhibitor with or without AR inhibition warrants investigation in the second-line endocrine therapy of metastatic ER+ BC.

However, the clinical development of enzalutamide and other AR inhibitors has been limited largely because of the inability to definitively identify the patients most likely to benefit from AR-targeted therapy. For these agents to have clinical utility in BC, predictive biomarkers are essential.

It is well-known that cancer may cause metabolic and physiological alterations that can affect the nutritional needs for carbohydrate, protein, vitamin, fat, and minerals [117]. Moreover, symptoms such as early satiety, anorexia, changes in taste and smell, and disturbances of the gastrointestinal tract are common side effects of antineoplastic treatment, and can lead to inadequate nutrient intake and consequent malnutrition [118,119]. Therefore, maintaining energy balance or preventing weight loss is fundamental. Nutritional screening and assessment for survivors should begin while treatment is being planned, and should focus on both the current nutritional status and anticipated symptoms related to treatment that could affect dietary intake [120]. During active cancer treatment, the overall goals of nutritional care for survivors should be to prevent or reverse nutrient deficiencies, to preserve lean body mass, to minimize nutrition-related side effects (such as nausea, decreased appetite or taste changes), and to maximize QoL. Recent studies confirm the benefit of dietary counseling during antineoplastic treatment for improving outcomes [121,122,123]. Providing short-term individualized nutritional support can improve appetite and dietary intake and decrease the cancer treatment-related toxicities [117].

Moreover, an increasing number of studies have examined the therapeutic value of exercise during cancer treatment [124,125,126]. Most of them have examined women with early-stage BC receiving adjuvant therapies. Existing evidence strongly suggests that exercise is safe, feasible, and can also improve physical functioning and some aspects of QoL during cancer treatment [122,123,124,125,126]. The decision regarding how to maintain or when to initiate physical activity should be individualized to the survivor’s condition and personal preferences. Likewise, resistance training programs may be helpful in hindering rapidly occurring adverse body composition changes (i.e., sarcopenic obesity and osteopenia) that may occur among some cancer patients who receive systemic therapy [127].

## 7. Conclusions

The management of HR+ mBC in the elderly is a challenge. The heterogeneity and complexity of elderly patients, and the lack of randomized clinical trials and validated guidelines on older patients, represent challenges to their optimal treatment.

We suggest performing a CGA on all patients over 70 in order to identify who might benefit from active therapies. It is therefore of fundamental importance that the therapeutic decision for elderly mBC patients be taken within a multidisciplinary team, and that close collaboration between oncologists and geriatricians is guaranteed. However, CGA is underused because it is long and difficult to apply in busy routine clinical practice. In this regard, there are other tools for evaluating the frailty of elderly patients that help in the choice of treatment, such as the CARG-BC score.

Interventions to improve the participation of older adults in cancer trials should also be implemented. This approach will allow older patients to receive the same treatment opportunities as younger patients.

## Figures and Tables

**Table 1:** Retrospective and real-world evidence on CDK 4/6 inhibitors in elderly patients.

Trial	No. of Patients	Median Age	CDK 4/6 Inhibitors	Results
American Flatiron Study [73,74]	1400	66 (20% >75 years)	Palbociclib (±Letrozole)	PFS and OS >75 y vs. <75 y
HeLLENIC Cooperative Oncology Group (HeCOG) [75]	365	61 (12% >75 years)	Palbociclib/ribociclib (±ET)	PFS (>75) 10.9 months as 1st line vs. 7.5 months as 2nd line Safety: similar (19% G3-4 adverse events)
CompLEEment–1 [76]	3246	58 (24% >65–75 years; 9.5% >75 years)	Ribociclib/letrozole	Safety and efficacy consistent with that seen in phase III trials
Kish et al. [77]	763	64 (50% <65 years; 20% >75 years)	Palbociclib/ET	Safety and efficacy consistent with Paloma 1 and 2 trials
PALOMAGE [71]	407	79 (15% older than 85 years)	Palbociclib (±ET)	Incidence of grade 3–4 AEs: 40% <80 years; 31% >80 years Dose reduction occurred in 23% of the patients; 30% of the patients older than 80 years

## Data Availability

Not applicable.

## References

[1] Łukasiewicz S., Czeczelewski M., Forma A., Baj J., Sitarz R., Stanisławek A. (2021). Breast Cancer-Epidemiology, Risk Factors, Classification, Prognostic Markers, and Current Treatment Strategies–An Updated Review. Cancers.

[2] Siegel R.L., Miller K.D., Fuchs H.E., Jemal A. (2022). Cancer statistics, 2022. CA Cancer J. Clin..

[3] Tesarova P. (2016). Specific Aspects of Breast Cancer Therapy of Elderly Women. BioMed Res. Int..

[4] Smith B.D., Smith G.L., Hurria A., Hortobagyi G.N., Buchholz T.A. (2009). Future of cancer incidence in the United States: Burdens upon an aging, changing nation. J. Clin. Oncol. Off. J. Am. Soc. Clin. Oncol..

[5] Biganzoli L., Battisti N.M.L., Wildiers H., McCartney A., Colloca G., Kunkler I.H., Cardoso M.J., Cheung K.L., de Glas N.A., Trimboli R.M. (2021). Updated recommendations regarding the management of older patients with breast cancer: A joint paper from the European Society of Breast Cancer Specialists (EUSOMA) and the International Society of Geriatric Oncology (SIOG). Lancet. Oncol..

[6] Polverelli N., Tura P., Battipaglia G., Malagola M., Bernardi S., Gandolfi L., Zollner T., Zanaglio C., Farina M., Morello E. (2020). Multidimensional geriatric assessment for elderly hematological patients (≥60 years) submitted to allogeneic stem cell transplantation. A French-Italian 10-year experience on 228 patients. Bone Marrow Transplant..

[7] Brunello A., Sandri R., Extermann M. (2009). Multidimensional geriatric evaluation for older cancer patients as a clinical and research tool. Cancer Treat. Rev..

[8] Benz C.C. (2008). Impact of aging on the biology of breast cancer. Crit. Rev. Oncol./Hematol..

[9] Tricarico D., Convertino A.S., Mehmeti I., Ranieri G., Leonetti F., Laface C., Zizzo N. (2022). Inflammatory Related Reactions in Humans and in Canine Breast Cancers, A Spontaneous Animal Model of Disease. Front. Pharmacol..

[10] Jatoi I., Miller A.B. (2003). Why is breast-cancer mortality declining?. Lancet. Oncol..

[11] Bouchardy C., Rapiti E., Fioretta G., Laissue P., Neyroud-Caspar I., Schäfer P., Kurtz J., Sappino A.P., Vlastos G. (2003). Undertreatment strongly decreases prognosis of breast cancer in elderly women. J. Clin. Oncol. Off. J. Am. Soc. Clin. Oncol..

[12] Ferraro E., Walsh E.M., Tao J.J., Chandarlapaty S., Jhaveri K. (2022). Accelerating drug development in breast cancer: New frontiers for ER inhibition. Cancer Treat. Rev..

[13] Davies C., Godwin J., Gray R., Clarke M., Cutter D., Darby S., McGale P., Pan H.C., Taylor C., Wang Y.C. (2011). Relevance of breast cancer hormone receptors and other factors to the efficacy of adjuvant tamoxifen: Patient-level meta-analysis of randomised trials. Lancet.

[14] Early Breast Cancer Trialists’ Collaborative Group (2015). Aromatase inhibitors versus tamoxifen in early breast cancer: Patient-level meta-analysis of the randomised trials. Lancet.

[15] Davies C., Pan H., Godwin J., Gray R., Arriagada R., Raina V., Abraham M., Medeiros Alencar V.H., Badran A., Bonfill X. (2013). Long-term effects of continuing adjuvant tamoxifen to 10 years versus stopping at 5 years after diagnosis of oestrogen receptor-positive breast cancer: ATLAS, a randomised trial. Lancet.

[16] Goss P.E., Ingle J.N., Pritchard K.I., Robert N.J., Muss H., Gralow J., Gelmon K., Whelan T., Strasser-Weippl K., Rubin S. (2016). Extending Aromatase-Inhibitor Adjuvant Therapy to 10 Years. N. Engl. J. Med..

[17] Mamounas E.P., Bandos H., Lembersky B.C., Jeong J.H., Geyer C.E., Rastogi P., Fehrenbacher L., Graham M.L., Chia S.K., Brufsky A.M. (2019). Use of letrozole after aromatase inhibitor-based therapy in postmenopausal breast cancer (NRG Oncology/NSABP B-42): A randomised, double-blind, placebo-controlled, phase 3 trial. Lancet. Oncol..

[18] Burstein H.J. (2020). Systemic Therapy for Estrogen Receptor-Positive, HER2-Negative Breast Cancer. N. Engl. J. Med..

[19] Robertson J.F.R., Bondarenko I.M., Trishkina E., Dvorkin M., Panasci L., Manikhas A., Shparyk Y., Cardona-Huerta S., Cheung K.L., Philco-Salas M.J. (2016). Fulvestrant 500 mg versus anastrozole 1 mg for hormone receptor-positive advanced breast cancer (FALCON): An international, randomised, double-blind, phase 3 trial. Lancet.

[20] Fanning S.W., Jeselsohn R., Dharmarajan V., Mayne C.G., Karimi M., Buchwalter G., Houtman R., Toy W., Fowler C.E., Han R. (2018). The SERM/SERD bazedoxifene disrupts ESR1 helix 12 to overcome acquired hormone resistance in breast cancer cells. Elife.

[21] Jeselsohn R., Yelensky R., Buchwalter G., Frampton G., Meric-Bernstam F., Gonzalez-Angulo A.M., Ferrer-Lozano J., Perez-Fidalgo J.A., Cristofanilli M., Gómez H. (2014). Emergence of constitutively active estrogen receptor-α mutations in pretreated advanced estrogen receptor-positive breast cancer. Clin. Cancer Res. Off. J. Am. Assoc. Cancer Res..

[22] Jeselsohn R., Buchwalter G., De Angelis C., Brown M., Schiff R. (2015). ESR1 mutations—A mechanism for acquired endocrine resistance in breast cancer. Nat. Reviews. Clin. Oncol..

[23] Niu J., Andres G., Kramer K., Kundranda M.N., Alvarez R.H., Klimant E., Parikh A.R., Tan B., Staren E.D., Markman M. (2015). Incidence and clinical significance of ESR1 mutations in heavily pretreated metastatic breast cancer patients. OncoTargets Ther..

[24] Freitag C.E., Mei P., Wei L., Parwani A.V., Li Z. (2021). ESR1 genetic alterations and their association with clinicopathologic characteristics in advanced breast cancer: A single academic institution experience. Hum. Pathol..

[25] Corti C., De Angelis C., Bianchini G., Malorni L., Giuliano M., Hamilton E., Jeselsohn R., Jhaveri K., Curigliano G., Criscitiello C. (2023). Novel endocrine therapies: What is next in estrogen receptor positive, HER2 negative breast cancer?. Cancer Treat. Rev..

[26] Finn R.S., Crown J.P., Lang I., Boer K., Bondarenko I.M., Kulyk S.O., Ettl J., Patel R., Pinter T., Schmidt M. (2015). The cyclin-dependent kinase 4/6 inhibitor palbociclib in combination with letrozole versus letrozole alone as first-line treatment of oestrogen receptor-positive, HER2-negative, advanced breast cancer (PALOMA-1/TRIO-18): A randomised phase 2 study. Lancet Oncol..

[27] Finn R.S., Martin M., Rugo H.S., Jones S., Im S.-A., Gelmon K., Harbeck N., Lipatov O.N., Walshe J.M., Moulder S. (2016). Palbociclib and Letrozole in Advanced Breast Cancer. N. Engl. J. Med..

[28] Turner N.C., Slamon D.J., Ro J., Bondarenko I., Im S.-A., Masuda N., Colleoni M., DeMichele A., Loi S., Verma S. (2018). Overall Survival with Palbociclib and Fulvestrant in Advanced Breast Cancer. N. Engl. J. Med..

[29] Hortobagyi G.N., Stemmer S.M., Burris H.A., Yap Y.-S., Sonke G.S., Hart L., Campone M., Petrakova K., Winer E.P., Janni W. (2022). Overall Survival with Ribociclib plus Letrozole in Advanced Breast Cancer. N. Engl. J. Med..

[30] Slamon D.J., Neven P., Chia S., Fasching P.A., De Laurentiis M., Im S.-A., Petrakova K., Bianchi G.V., Esteva F.J., Martín M. (2019). Overall Survival with Ribociclib plus Fulvestrant in Advanced Breast Cancer. N. Engl. J. Med..

[31] Goetz M.P., Toi M., Campone M., Sohn J., Paluch-Shimon S., Huober J., Park I.H., Trédan O., Chen S.C., Manso L. (2017). MONARCH 3: Abemaciclib As Initial Therapy for Advanced Breast Cancer. J. Clin. Oncol. Off. J. Am. Soc. Clin. Oncol..

[32] Sledge G.W., Toi M., Neven P., Sohn J., Inoue K., Pivot X., Burdaeva O., Okera M., Masuda N., Kaufman P.A. (2017). MONARCH 2: Abemaciclib in Combination with Fulvestrant in Women with HR+/HER2− Advanced Breast Cancer Who Had Progressed While Receiving Endocrine Therapy. J. Clin. Oncol. Off. J. Am. Soc. Clin. Oncol..

[33] Beaver J.A., Park B.H. (2012). The BOLERO-2 trial: The addition of everolimus to exemestane in the treatment of postmenopausal hormone receptor-positive advanced breast cancer. Future Oncol..

[34] Robson M., Im S.-A., Senkus E., Xu B., Domchek S.M., Masuda N., Delaloge S., Li W., Tung N., Armstrong A. (2017). Olaparib for Metastatic Breast Cancer in Patients with a Germline BRCA Mutation. N. Engl. J. Med..

[35] Litton J.K., Rugo H.S., Ettl J., Hurvitz S.A., Gonçalves A., Lee K.-H., Fehrenbacher L., Yerushalmi R., Mina L.A., Martin M. (2018). Talazoparib in Patients with Advanced Breast Cancer and a Germline BRCA Mutation. N. Engl. J. Med..

[36] Bidard F.C., Kaklamani V.G., Neven P., Streich G., Montero A.J., Forget F., Mouret-Reynier M.A., Sohn J.H., Taylor D., Harnden K.K. (2022). Elacestrant (oral selective estrogen receptor degrader) Versus Standard Endocrine Therapy for Estrogen Receptor-Positive, Human Epidermal Growth Factor Receptor 2-Negative Advanced Breast Cancer: Results from the Randomized Phase III EMERALD Trial. J. Clin. Oncol. Off. J. Am. Soc. Clin. Oncol..

[37] Rose S. (2023). Second Oral SERD Shines in ER+ Breast Cancer. Cancer Discov..

[38] Oliveira M., Pominchuk D., Hamilton E.P., Kulyaba Y., Melkadze T., Neven P., Nemsadze G., Andabekov T., Semegen Y., Hotko Y. (2023). Clinical activity of camizestrant, a next-generation SERD, versus fulvestrant in patients with a detectable ESR1 mutation: Exploratory analysis of the SERENA-2 phase 2 trial. J. Clin. Oncol..

[39] André F., Ciruelos E., Rubovszky G., Campone M., Loibl S., Rugo H.S., Iwata H., Conte P., Mayer I.A., Kaufman B. (2019). Alpelisib for PIK3CA-Mutated, Hormone Receptor-Positive Advanced Breast Cancer. N. Engl. J. Med..

[40] André F., Ciruelos E.M., Juric D., Loibl S., Campone M., Mayer I.A., Rubovszky G., Yamashita T., Kaufman B., Lu Y.S. (2021). Alpelisib plus fulvestrant for PIK3CA-mutated, hormone receptor-positive, human epidermal growth factor receptor-2-negative advanced breast cancer: Final overall survival results from SOLAR-1. Ann. Oncol. Off. J. Eur. Soc. Med. Oncol..

[41] Rugo H.S., Lerebours F., Ciruelos E., Drullinsky P., Ruiz-Borrego M., Neven P., Park Y.H., Prat A., Bachelot T., Juric D. (2021). Alpelisib plus fulvestrant in *PIK3CA*-mutated, hormone receptor-positive advanced breast cancer after a CDK4/6 inhibitor (BYLieve): One cohort of a phase 2, multicentre, open-label, non-comparative study. Lancet Oncol..

[42] Patruno R., Passantino G., Laface C., Tinelli A., Zito A., Ruggieri R., Luposella F., Gadaleta P., Laforgia M., Lacitignola L. (2020). Microvascular Density, Endothelial Area, and Ki-67 Proliferative Index Correlate Each Other in Cat Post-Injection Fibrosarcoma. Cells.

[43] Modi S., Jacot W., Yamashita T., Sohn J., Vidal M., Tokunaga E., Tsurutani J., Ueno N.T., Prat A., Chae Y.S. (2022). Trastuzumab Deruxtecan in Previously Treated HER2-Low Advanced Breast Cancer. N. Engl. J. Med..

[44] Rugo H.S., Bardia A., Tolaney S.M., Arteaga C., Cortes J., Sohn J., Marmé F., Hong Q., Delaney R.J., Hafeez A. (2020). TROPiCS-02: A Phase III study investigating sacituzumab govitecan in the treatment of HR+/HER2− metastatic breast cancer. Future Oncol..

[45] Liu J., Ma J., Liu Y., Xia J., Li Y., Wang Z.P., Wei W. (2020). PROTACs: A novel strategy for cancer therapy. Semin. Cancer Biol..

[46] Puyang X., Furman C., Zheng G.Z., Wu Z.J., Banka D., Aithal K., Agoulnik S., Bolduc D.M., Buonamici S., Caleb B. (2018). Discovery of Selective Estrogen Receptor Covalent Antagonists for the Treatment of ERα(WT) and ERα(MUT) Breast Cancer. Cancer Discov..

[47] Depboylu B. (2020). Treatment and patient related quality of life issues in elderly and very elderly breast cancer patients. Transl. Cancer Res..

[48] Presley C.J., Krok-Schoen J.L., Wall S.A., Noonan A.M., Jones D.C., Folefac E., Williams N., Overcash J., Rosko A.E. (2020). Implementing a multidisciplinary approach for older adults with Cancer: Geriatric oncology in practice. BMC Geriatr..

[49] Wildiers H., Heeren P., Puts M., Topinkova E., Janssen-Heijnen M.L., Extermann M., Falandry C., Artz A., Brain E., Colloca G. (2014). International Society of Geriatric Oncology consensus on geriatric assessment in older patients with cancer. J. Clin. Oncol. Off. J. Am. Soc. Clin. Oncol..

[50] Carreca I., Balducci L., Extermann M. (2005). Cancer in the older person. Cancer Treat. Rev..

[51] Loh K.P., Soto-Perez-de-Celis E., Hsu T., de Glas N.A., Battisti N.M.L., Baldini C., Rodrigues M., Lichtman S.M., Wildiers H. (2018). What Every Oncologist Should Know about Geriatric Assessment for Older Patients with Cancer: Young International Society of Geriatric Oncology Position Paper. J. Oncol. Pract..

[52] Hutchins L.F., Unger J.M., Crowley J.J., Coltman C.A., Albain K.S. (1999). Underrepresentation of patients 65 years of age or older in cancer-treatment trials. N. Engl. J. Med..

[53] Ludmir E.B., Mainwaring W., Lin T.A., Miller A.B., Jethanandani A., Espinoza A.F., Mandel J.J., Lin S.H., Smith B.D., Smith G.L. (2019). Factors Associated with Age Disparities Among Cancer Clinical Trial Participants. JAMA Oncol..

[54] Carleton N., Nasrazadani A., Gade K., Beriwal S., Barry P.N., Brufsky A.M., Bhargava R., Berg W.A., Zuley M.L., van Londen G.J. (2022). Personalising therapy for early-stage oestrogen receptor-positive breast cancer in older women. Lancet. Healthy Longev..

[55] Balducci L., Extermann M. (2000). Management of cancer in the older person: A practical approach. Oncologist.

[56] Bernabei R., Venturiero V., Tarsitani P., Gambassi G. (2000). The comprehensive geriatric assessment: When, where, how. Crit. Rev. Oncol./Hematol..

[57] Zagonel V. (2001). Importance of a comprehensive geriatric assessment in older cancer patients. Eur. J. Cancer.

[58] Basso U., Monfardini S. (2004). Multidimensional geriatric evaluation in elderly cancer patients: A practical approach. Eur. J. Cancer Care.

[59] Magnuson A., Sedrak M.S., Gross C.P., Tew W.P., Klepin H.D., Wildes T.M., Muss H.B., Dotan E., Freedman R.A., O’Connor T. (2021). Development and Validation of a Risk Tool for Predicting Severe Toxicity in Older Adults Receiving Chemotherapy for Early-Stage Breast Cancer. J. Clin. Oncol..

[60] Abdel-Razeq H., Abu Rous F., Abuhijla F., Abdel-Razeq N., Edaily S. (2022). Breast Cancer in Geriatric Patients: Current Landscape and Future Prospects. Clin. Interv. Aging.

[61] Gennari R., Curigliano G., Rotmensz N., Robertson C., Colleoni M., Zurrida S., Nolè F., de Braud F., Orlando L., Leonardi M.C. (2004). Breast carcinoma in elderly women: Features of disease presentation, choice of local and systemic treatments compared with younger postmenopasual patients. Cancer.

[62] Spazzapan S., Crivellari D., Bedard P., Lombardi D., Miolo G., Scalone S., Veronesi A. (2011). Therapeutic management of breast cancer in the elderly. Expert Opin. Pharmacother..

[63] Orimo H. (2006). Reviewing the definition of elderly. Nihon Ronen Igakkai Zasshi. Jpn. J. Geriatr..

[64] Baselga J., Campone M., Piccart M., Burris H.A., Rugo H.S., Sahmoud T., Noguchi S., Gnant M., Pritchard K.I., Lebrun F. (2012). Everolimus in postmenopausal hormone-receptor-positive advanced breast cancer. N. Engl. J. Med..

[65] Beck J.T., Hortobagyi G.N., Campone M., Lebrun F., Deleu I., Rugo H.S., Pistilli B., Masuda N., Hart L., Melichar B. (2014). Everolimus plus exemestane as first-line therapy in HR⁺, HER2^−^ advanced breast cancer in BOLERO-2. Breast Cancer Res. Treat..

[66] Perrone M., Giuliani F., Sanna V., Bruno S., Melaccio A., Santoro A.N., Laface C., Maselli F.M., Iaia M.L., Guarini C. (2023). Advances in pharmacotherapies that target the cell cycle for treatment of breast cancer: Where are we at today?. Expert Opin. Pharmacother..

[67] Finn R.S., Boer K., Bondarenko I., Patel R., Pinter T., Schmidt M., Shparyk Y.V., Thummala A., Voitko N., Bananis E. (2020). Overall survival results from the randomized phase 2 study of palbociclib in combination with letrozole versus letrozole alone for first-line treatment of ER+/HER2− advanced breast cancer (PALOMA-1, TRIO-18). Breast Cancer Res. Treat..

[68] Rugo H.S., Turner N.C., Finn R.S., Joy A.A., Verma S., Harbeck N., Masuda N., Im S.A., Huang X., Kim S. (2018). Palbociclib plus endocrine therapy in older women with HR+/HER2− advanced breast cancer: A pooled analysis of randomised PALOMA clinical studies. Eur. J. Cancer.

[69] Sonke G.S., Hart L.L., Campone M., Erdkamp F., Janni W., Verma S., Villanueva C., Jakobsen E., Alba E., Wist E. (2018). Ribociclib with letrozole vs letrozole alone in elderly patients with hormone receptor-positive, HER2-negative breast cancer in the randomized MONALEESA-2 trial. Breast Cancer Res. Treat..

[70] Goetz M.P., Okera M., Wildiers H., Campone M., Grischke E.M., Manso L., André V.A.M., Chouaki N., San Antonio B., Toi M. (2021). Safety and efficacy of abemaciclib plus endocrine therapy in older patients with hormone receptor-positive/human epidermal growth factor receptor 2-negative advanced breast cancer: An age-specific subgroup analysis of MONARCH 2 and 3 trials. Breast Cancer Res. Treat..

[71] Caillet P., Pulido M., Brain E., Falandry C., Desmoulins I., Ghebriou D., Soubeyran P.-L., Paillaud E., Rifi N., Vauthier J.M. (2021). PALOMAGE, a French real-world cohort of elderly women beyond age 70 with advanced breast cancer receiving palbociclib: Baseline characteristics and safety evaluation. J. Clin. Oncol..

[72] Battisti N.M.L., De Glas N., Sedrak M.S., Loh K.P., Liposits G., Soto-Perez-de-Celis E., Krok-Schoen J.L., Menjak I.B., Ring A. (2018). Use of cyclin-dependent kinase 4/6 (CDK4/6) inhibitors in older patients with ER-positive HER2-negative breast cancer: Young International Society of Geriatric Oncology review paper. Ther. Adv. Med. Oncol..

[73] Patt D., Liu X., Li B., McRoy L., Layman R.M., Brufsky A. (2022). Real-World Treatment Patterns and Outcomes of Palbociclib Plus an Aromatase Inhibitor for Metastatic Breast Cancer: Flatiron Database Analysis. Clin. Breast Cancer.

[74] DeMichele A., Cristofanilli M., Brufsky A., Liu X., Mardekian J., McRoy L., Layman R.M., Rugo H.S., Finn R.S. (2020). Abstract P1-19-02: Overall survival for first-line palbociclib plus letrozole vs letrozole alone for HR+/HER2- metastatic breast cancer patients in US real-world clinical practice. Cancer Res..

[75] Fountzilas E., Koliou G.A., Vozikis A., Rapti V., Nikolakopoulos A., Boutis A., Christopoulou A., Kontogiorgos I., Karageorgopoulou S., Lalla E. (2020). Real-world clinical outcome and toxicity data and economic aspects in patients with advanced breast cancer treated with cyclin-dependent kinase 4/6 (CDK4/6) inhibitors combined with endocrine therapy: The experience of the Hellenic Cooperative Oncology Group. ESMO Open.

[76] De Laurentiis M., Borstnar S., Campone M., Warner E., Bofill J.S., Jacot W., Dent S., Martin M., Ring A., Cottu P. (2021). Full population results from the core phase of CompLEEment-1, a phase 3b study of ribociclib plus letrozole as first-line therapy for advanced breast cancer in an expanded population. Breast Cancer Res. Treat..

[77] Kish J.K., Ward M.A., Garofalo D., Ahmed H.V., McRoy L., Laney J., Zanotti G., Braverman J., Yu H., Feinberg B.A. (2018). Real-world evidence analysis of palbociclib prescribing patterns for patients with advanced/metastatic breast cancer treated in community oncology practice in the USA one year post approval. Breast Cancer Res. BCR.

[78] Turner N.C., Oliveira M., Howell S.J., Dalenc F., Cortes J., Gomez Moreno H.L., Hu X., Jhaveri K., Krivorotko P., Loibl S. (2023). Capivasertib in Hormone Receptor–Positive Advanced Breast Cancer. New Engl. J. Med..

[79] Varghese F., Wong J. (2018). Breast Cancer in the Elderly. Surg. Clin. North Am..

[80] Soto-Perez-de-Celis E., Li D., Yuan Y., Lau Y.M., Hurria A. (2018). Functional versus chronological age: Geriatric assessments to guide decision making in older patients with cancer. Lancet. Oncol..

[81] Cardoso F., Bedard P.L., Winer E.P., Pagani O., Senkus-Konefka E., Fallowfield L.J., Kyriakides S., Costa A., Cufer T., Albain K.S. (2009). International guidelines for management of metastatic breast cancer: Combination vs. sequential single-agent chemotherapy. J. Natl. Cancer Inst..

[82] Okouneva T., Azarenko O., Wilson L., Littlefield B.A., Jordan M.A. (2008). Inhibition of centromere dynamics by eribulin (E7389) during mitotic metaphase. Mol. Cancer Ther..

[83] Smith J.A., Wilson L., Azarenko O., Zhu X., Lewis B.M., Littlefield B.A., Jordan M.A. (2010). Eribulin binds at microtubule ends to a single site on tubulin to suppress dynamic instability. Biochemistry.

[84] Cortes J., O’Shaughnessy J., Loesch D., Blum J.L., Vahdat L.T., Petrakova K., Chollet P., Manikas A., Diéras V., Delozier T. (2011). Eribulin monotherapy versus treatment of physician’s choice in patients with metastatic breast cancer (EMBRACE): A phase 3 open-label randomised study. Lancet.

[85] Vahdat L.T., Pruitt B., Fabian C.J., Rivera R.R., Smith D.A., Tan-Chiu E., Wright J., Tan A.R., Dacosta N.A., Chuang E. (2009). Phase II study of eribulin mesylate, a halichondrin B analog, in patients with metastatic breast cancer previously treated with an anthracycline and a taxane. J. Clin. Oncol. Off. J. Am. Soc. Clin. Oncol..

[86] Cortes J., Vahdat L., Blum J.L., Twelves C., Campone M., Roché H., Bachelot T., Awada A., Paridaens R., Goncalves A. (2010). Phase II study of the halichondrin B analog eribulin mesylate in patients with locally advanced or metastatic breast cancer previously treated with an anthracycline, a taxane, and capecitabine. J. Clin. Oncol. Off. J. Am. Soc. Clin. Oncol..

[87] Muss H., Cortes J., Vahdat L.T., Cardoso F., Twelves C., Wanders J., Dutcus C.E., Yang J., Seegobin S., O’Shaughnessy J. (2014). Eribulin monotherapy in patients aged 70 years and older with metastatic breast cancer. Oncol..

[88] Leo S., Arnoldi E., Repetto L., Coccorullo Z., Cinieri S., Fedele P., Cazzaniga M., Lorusso V., Latorre A., Campanella G. (2019). Eribulin Mesylate as Third or Subsequent Line Chemotherapy for Elderly Patients with Locally Recurrent or Metastatic Breast Cancer: A Multicentric Observational Study of GIOGer (Italian Group of Geriatric Oncology)-ERIBE. Oncol..

[89] Gradishar W.J., Tjulandin S., Davidson N., Shaw H., Desai N., Bhar P., Hawkins M., O’Shaughnessy J. (2005). Phase III trial of nanoparticle albumin-bound paclitaxel compared with polyethylated castor oil-based paclitaxel in women with breast cancer. J. Clin. Oncol. Off. J. Am. Soc. Clin. Oncol..

[90] Gradishar W.J., Krasnojon D., Cheporov S., Makhson A.N., Manikhas G.M., Clawson A., Bhar P. (2009). Significantly longer progression-free survival with nab-paclitaxel compared with docetaxel as first-line therapy for metastatic breast cancer. J. Clin. Oncol. Off. J. Am. Soc. Clin. Oncol..

[91] Liu Y., Ye G., Yan D., Zhang L., Fan F., Feng J. (2017). Role of nab-paclitaxel in metastatic breast cancer: A meta-analysis of randomized clinical trials. Oncotarget.

[92] Biganzoli L., Cinieri S., Berardi R., Pedersini R., McCartney A., Minisini A.M., Caremoli E.R., Spazzapan S., Magnolfi E., Brunello A. (2020). EFFECT: A randomized phase II study of efficacy and impact on function of two doses of nab-paclitaxel as first-line treatment in older women with advanced breast cancer. Breast Cancer Res. BCR.

[93] Koster K.L., Huober J., Joerger M. (2022). New antibody-drug conjugates (ADCs) in breast cancer-an overview of ADCs recently approved and in later stages of development. Explor. Target. Anti-Tumor Ther..

[94] Verma S., Miles D., Gianni L., Krop I.E., Welslau M., Baselga J., Pegram M., Oh D.Y., Diéras V., Guardino E. (2012). Trastuzumab emtansine for HER2-positive advanced breast cancer. N. Engl. J. Med..

[95] von Minckwitz G., Huang C.S., Mano M.S., Loibl S., Mamounas E.P., Untch M., Wolmark N., Rastogi P., Schneeweiss A., Redondo A. (2019). Trastuzumab Emtansine for Residual Invasive HER2-Positive Breast Cancer. N. Engl. J. Med..

[96] André F., Hee Park Y., Kim S.B., Takano T., Im S.A., Borges G., Lima J.P., Aksoy S., Gavila Gregori J., De Laurentiis M. (2023). Trastuzumab deruxtecan versus treatment of physician’s choice in patients with HER2-positive metastatic breast cancer (DESTINY-Breast02): A randomised, open-label, multicentre, phase 3 trial. Lancet.

[97] Goldenberg D.M., Cardillo T.M., Govindan S.V., Rossi E.A., Sharkey R.M. (2015). Trop-2 is a novel target for solid cancer therapy with sacituzumab govitecan (IMMU-132), an antibody-drug conjugate (ADC). Oncotarget.

[98] Bardia A., Mayer I.A., Vahdat L.T., Tolaney S.M., Isakoff S.J., Diamond J.R., O’Shaughnessy J., Moroose R.L., Santin A.D., Abramson V.G. (2019). Sacituzumab Govitecan-hziy in Refractory Metastatic Triple-Negative Breast Cancer. N. Engl. J. Med..

[99] Bardia A., Tolaney S.M., Punie K., Loirat D., Oliveira M., Kalinsky K., Zelnak A., Aftimos P., Dalenc F., Sardesai S. (2021). Biomarker analyses in the phase III ASCENT study of sacituzumab govitecan versus chemotherapy in patients with metastatic triple-negative breast cancer. Ann. Oncol. Off. J. Eur. Soc. Med. Oncol..

[100] Tolaney S.M., Bardia A., Marmé F., Cortés J., Schmid P., Loirat D., Tredan O., Ciruelos E.M., Dalenc F., Gómez Pardo P. (2023). Final overall survival (OS) analysis from the phase 3 TROPiCS-02 study of sacituzumab govitecan (SG) in patients (pts) with hormone receptor–positive/HER2-negative (HR+/HER2−) metastatic breast cancer (mBC). J. Clin. Oncol..

[101] Brufsky A.M. (2017). Long-term management of patients with hormone receptor-positive metastatic breast cancer: Concepts for sequential and combination endocrine-based therapies. Cancer Treat. Rev..

[102] Rechoum Y., Rovito D., Iacopetta D., Barone I., Andò S., Weigel N.L., O’Malley B.W., Brown P.H., Fuqua S.A. (2014). AR collaborates with ERα in aromatase inhibitor-resistant breast cancer. Breast Cancer Res. Treat..

[103] Takagi K., Miki Y., Nagasaki S., Hirakawa H., Onodera Y., Akahira J., Ishida T., Watanabe M., Kimijima I., Hayashi S. (2010). Increased intratumoral androgens in human breast carcinoma following aromatase inhibitor exemestane treatment. Endocr.-Relat. Cancer.

[104] Collins L.C., Cole K.S., Marotti J.D., Hu R., Schnitt S.J., Tamimi R.M. (2011). Androgen receptor expression in breast cancer in relation to molecular phenotype: Results from the Nurses’ Health Study. Mod. Pathol. Off. J. United States Can. Acad. Pathol. Inc.

[105] Loibl S., Müller B.M., von Minckwitz G., Schwabe M., Roller M., Darb-Esfahani S., Ataseven B., du Bois A., Fissler-Eckhoff A., Gerber B. (2011). Androgen receptor expression in primary breast cancer and its predictive and prognostic value in patients treated with neoadjuvant chemotherapy. Breast Cancer Res. Treat..

[106] Ricciardelli C., Bianco-Miotto T., Jindal S., Butler L.M., Leung S., McNeil C.M., O’Toole S.A., Ebrahimie E., Millar E.K.A., Sakko A.J. (2018). The Magnitude of Androgen Receptor Positivity in Breast Cancer Is Critical for Reliable Prediction of Disease Outcome. Clin. Cancer Res. Off. J. Am. Assoc. Cancer Res..

[107] Vera-Badillo F.E., Templeton A.J., de Gouveia P., Diaz-Padilla I., Bedard P.L., Al-Mubarak M., Seruga B., Tannock I.F., Ocana A., Amir E. (2014). Androgen receptor expression and outcomes in early breast cancer: A systematic review and meta-analysis. J. Natl. Cancer Inst..

[108] De Amicis F., Thirugnansampanthan J., Cui Y., Selever J., Beyer A., Parra I., Weigel N.L., Herynk M.H., Tsimelzon A., Lewis M.T. (2010). Androgen receptor overexpression induces tamoxifen resistance in human breast cancer cells. Breast Cancer Res. Treat..

[109] D’Amato N.C., Gordon M.A., Babbs B., Spoelstra N.S., Carson Butterfield K.T., Torkko K.C., Phan V.T., Barton V.N., Rogers T.J., Sartorius C.A. (2016). Cooperative Dynamics of AR and ER Activity in Breast Cancer. Mol. Cancer Res. MCR.

[110] Scher H.I., Fizazi K., Saad F., Taplin M.E., Sternberg C.N., Miller K., de Wit R., Mulders P., Chi K.N., Shore N.D. (2012). Increased survival with enzalutamide in prostate cancer after chemotherapy. N. Engl. J. Med..

[111] Beer T.M., Armstrong A.J., Rathkopf D.E., Loriot Y., Sternberg C.N., Higano C.S., Iversen P., Bhattacharya S., Carles J., Chowdhury S. (2014). Enzalutamide in metastatic prostate cancer before chemotherapy. N. Engl. J. Med..

[112] Hussain M., Fizazi K., Saad F., Rathenborg P., Shore N., Ferreira U., Ivashchenko P., Demirhan E., Modelska K., Phung D. (2018). Enzalutamide in Men with Nonmetastatic, Castration-Resistant Prostate Cancer. N. Engl. J. Med..

[113] Armstrong A.J., Szmulewitz R.Z., Petrylak D.P., Holzbeierlein J., Villers A., Azad A., Alcaraz A., Alekseev B., Iguchi T., Shore N.D. (2019). ARCHES: A Randomized, Phase III Study of Androgen Deprivation Therapy With Enzalutamide or Placebo in Men with Metastatic Hormone-Sensitive Prostate Cancer. J. Clin. Oncol. Off. J. Am. Soc. Clin. Oncol..

[114] Davis I.D., Martin A.J., Stockler M.R., Begbie S., Chi K.N., Chowdhury S., Coskinas X., Frydenberg M., Hague W.E., Horvath L.G. (2019). Enzalutamide with Standard First-Line Therapy in Metastatic Prostate Cancer. N. Engl. J. Med..

[115] Krop I., Abramson V., Colleoni M., Traina T., Holmes F., Garcia-Estevez L., Hart L., Awada A., Zamagni C., Morris P.G. (2020). A Randomized Placebo Controlled Phase II Trial Evaluating Exemestane with or without Enzalutamide in Patients with Hormone Receptor–Positive Breast Cancer. Clin. Cancer Res..

[116] Elias A.D., Spoelstra N.S., Staley A.W., Sams S., Crump L.S., Vidal G.A., Borges V.F., Kabos P., Diamond J.R., Shagisultanova E. (2023). Phase II trial of fulvestrant plus enzalutamide in ER+/HER2− advanced breast cancer. npj Breast Cancer.

[117] Doyle C., Kushi L.H., Byers T., Courneya K.S., Demark-Wahnefried W., Grant B., McTiernan A., Rock C.L., Thompson C., Gansler T. (2006). Nutrition and Physical Activity During and After Cancer Treatment: An American Cancer Society Guide for Informed Choices. CA Cancer J. Clin..

[118] Ollenschlaeger G., Konkol K., Wickramanayake P.D., Schrappe-Baecher M., Mueller J.M. (1989). Nutrient intake and nitrogen metabolism in cancer patients during oncological chemotherapy. Am. J. Clin. Nutr..

[119] Nitenberg G., Raynard B. (2000). Nutritional support of the cancer patient: Issues and dilemmas. Crit. Rev. Oncol./Hematol..

[120] McMahon K., Brown J.K. (2000). Nutritional screening and assessment. Semin. Oncol. Nurs..

[121] Ravasco P., Monteiro-Grillo I., Vidal P.M., Camilo M.E. (2005). Dietary counseling improves patient outcomes: A prospective, randomized, controlled trial in colorectal cancer patients undergoing radiotherapy. J. Clin. Oncol. Off. J. Am. Soc. Clin. Oncol..

[122] Rock C.L. (2005). Dietary counseling is beneficial for the patient with cancer. J. Clin. Oncol. Off. J. Am. Soc. Clin. Oncol..

[123] McGough C., Baldwin C., Frost G., Andreyev H.J. (2004). Role of nutritional intervention in patients treated with radiotherapy for pelvic malignancy. Br. J. Cancer.

[124] Courneya K.S. (2003). Exercise in cancer survivors: An overview of research. Med. Sci. Sports Exerc..

[125] Schmitz K.H., Holtzman J., Courneya K.S., Mâsse L.C., Duval S., Kane R. (2005). Controlled physical activity trials in cancer survivors: A systematic review and meta-analysis. Cancer Epidemiol. Biomark. Prev. Publ. Am. Assoc. Cancer Res. Cosponsored Am. Soc. Prev. Oncol..

[126] Knols R., Aaronson N.K., Uebelhart D., Fransen J., Aufdemkampe G. (2005). Physical exercise in cancer patients during and after medical treatment: A systematic review of randomized and controlled clinical trials. J. Clin. Oncol. Off. J. Am. Soc. Clin. Oncol..

[127] Demark-Wahnefried W., Kenyon A.J., Eberle P., Skye A., Kraus W.E. (2002). Preventing sarcopenic obesity among breast cancer patients who receive adjuvant chemotherapy: Results of a feasibility study. Clin. Exerc. Physiol..

